# Neurotensin and its high affinity receptor 1 as a potential pharmacological target in cancer therapy

**DOI:** 10.3389/fendo.2012.00184

**Published:** 2013-01-17

**Authors:** Zherui Wu, Daniel Martinez-Fong, Jean Trédaniel, Patricia Forgez

**Affiliations:** ^1^INSERM-UPMC UMR_S938, Hôpital Saint-AntoineParis, France; ^2^Departamento de Fisiologïa, Biofïsica y Neurociencias, Centro de Investigación y de Estudios Avanzados del Instituto Politécnico NacionalMexico City, Mexico; ^3^Unité de Cancérologie Thoracique, Groupe Hospitalier Paris Saint-Joseph/Université Paris DescartesParis, France

**Keywords:** cancer therapy, cancer progression, carcinogenesis, neurotensin, neurotensin receptor

## Abstract

Cancer is a worldwide health problem. Personalized treatment represents a future advancement for cancer treatment, in part due to the development of targeted therapeutic drugs. These molecules are expected to be more effective than current treatments and less harmful to normal cells. The discovery and validation of new targets are the foundation and the source of these new therapies. The neurotensinergic system has been shown to enhance cancer progression in various cancers such as pancreatic, prostate, lung, breast, and colon cancer. It also triggers multiple oncogenic signaling pathways, such as the PKC/ERK and AKT pathways. In this review, we discuss the contribution of the neurotensinergic system to cancer progression, as well as the regulation and mechanisms of the system in order to highlight its potential as a therapeutic target, and its prospect for its use as a treatment in certain cancers.

## INTRODUCTION

Cancer is one of the first, if not the first cause, of death in the world. Conventional treatment methods include surgery, radiotherapy, and chemotherapy. In many cases, only a supportive treatment can be offered to the patient. The last few decades have been marked by the accumulation of knowledge about the inner workings of the normal and cancer cell. Thus arose the therapeutic arsenal against cancer, and the so-called targeted biological therapies. Due to these new drugs, great progress has recently been achieved in the treatment of cancers considered refractory to previous therapies, including cancers of the liver, kidney, and melanoma skin. But other tumors, such as breast and lung cancer, have also greatly benefited from these advances. However, remissions are often transient and do not yet provide definitive cures of the patient. In this context the neurotensin system is proposed to be a candidate for therapeutical development.

Neurotensin (NTS) is a tridecapeptide originally isolated from calf hypothalamus (Carraway and Leeman, 1973). It acts as neurotransmitter or neuromodulator in the central nervous system and as a local hormone in the periphery, mainly the gastrointestinal tract (Vincent et al., 1999). In the brain, NTS modulates dopaminergic transmission in the nigrostriatal and mesocorticolimbic pathways as well as hormone secretion from the anterior pituitary (Vincent et al., 1999). It also exerts potent hypothermic and analgesic effects when injected into the central nervous system (Popp et al., 2007). NTS has also been related to central nervous system pathology such as Parkinson disease and schizophrenia (St-Gelais et al., 2006; Mustain et al., 2011). In the periphery, NTS is released by endocrine-like N cells predominantly in the small intestine (Reinecke, 1985). NTS has a dual function, as a paracrine and endocrine modulator of the digestive tract and as a growth factor on a variety of normal or cancer cells (Vincent et al., 1999; Carraway and Plona, 2006). The NTS/NTSR1 complex has been proposed to contribute to cancer progression because of the various oncogenic effects induced by NTS in tumors and in cancer cells from diverse origins (Thomas et al., 2003; Dupouy et al., 2011). In this review, we elaborate on how the NTS/NTSR1 complex could be developed as a possible target for cancer therapy.

## NEUROTENSIN/NEUROTENSIN RECEPTOR COMPLEX

### NEUROTENSINERGIC SYSTEM

The biological effects of NTS are known to be mediated through three receptors, two G protein-coupled receptors, NTSR1 and NTSR2, and a single transmembrane domain sorting receptor, the NTSR3 (Vincent et al., 1999; Mazella and Vincent, 2006). NTSR3 is a member of the receptor family related to the yeast sorting receptor Vps10p (Petersen et al., 1997).

#### Neurotensin receptor 1, NTSR1

The effects of NTS are primarily transmitted through its high affinity receptor, the NTSR1 which has a sub-nanomolar affinity for NTS. This 424 amino acid receptor has been identified in the brain and in various cancer cells (Tanaka et al., 1990). The signaling pathways induced by the NTS/NTSR1 complex have been studied in different cellular types, such as N1E-115, HT-29, and NTSR1-transfected CHO overexpressing NTSR1. The stimulation of NTSR1 by its ligand (NTS) leads to activation of phospholipase C (PLC) via its coupling to the prime Gαq/11 subunit (Wang and Wu, 1996; Najimi et al., 2002). The activation of PLC leads to the production of inositol triphosphate (IP3) and diacylglycerol from membrane phospholipids (PIP2). These two second messengers induce the activation of PKC and the mobilization of intracellular calcium which are key oncogenic effectors (Bozou et al., 1986; Snider et al., 1986; Turner et al., 1990).

Several signaling pathways potentially involved in cell proliferation, survival, migration, and invasion are described after NTSR1 stimulation. The signaling mechanisms mediating the effects of neurotensinergic system involve multiple pathways and are cell-dependent. The NTS-induced PKC/ERK signaling pathway is the most well studied. The PKC activation stimulated by NTS/NTSR1 was demonstrated by using broad isotype inhibitors, such as Gö6976 which specifically inhibit the conventional PKCs α and β1 in CHO-NTSR1-transfected cells and in endogenously NTSR1-expressing cells from colon, lung, and pancreatic cells (Poinot-Chazel et al., 1996; Seufferlein and Rozengurt, 1996; Ehlers et al., 2000; Muller et al., 2011). The NTSR1 contribution in this activation was confirmed by its inhibition by a specific NTSR1 antagonist, the SR 489692 (Gully et al., 1993). PKC activation induce mitogen-activated protein kinase (MAPK) via direct stimulation of Raf-1, independently of Ras, or by transactivation of the epidermal growth factor receptor (EGFR) (Guha et al., 2003). EGFR transactivation by NTS/NTSR1 complex has been observed in several cell lines. In prostatic cancer PC3 cell line, NTS activates mitogenesis through EGFR transactivation in a PKC-dependent pathway, and the stimulation of the Raf–MEK–ERK. This effect was also PI3 kinase (PI3K)-dependent (Hassan et al., 2004). NTS also induces a time-dependent increase in Tyr_845_ EGFR phosphorylation, c-Src phosphorylation and signal transducer. NTS is also an activator of transcription 5b (Stat5b), a downstream effector of Tyr_845_ EGFR phosphorylated (Amorino et al., 2007). In colonic HT-29 cells, the EGFR tyrosine kinase inhibitor, gefitinib, blocks NTS-stimulated phosphorylation of both MAPK and Akt, indicating the transactivation of EGFR independently of PKC activation. However, in the colonic HCT116 cells, NTS/NTSR1 induces a PKC-dependent MAPK phosphorylation and an EGFR metalloproteinase-mediated transactivation, that is associated with a gefitinib-sensitive phosphorylation of the downstream adaptor protein Shc. The activation of Akt is only partly inhibited by gefitinib, suggesting an additional mechanism to EGFR transactivation (Muller et al., 2011). The mechanism of NTS-induced EGFR transactivation is still not clearly elucidated. The release of EGFR ligands-like (TGF-α, Hb-EGF, or amphiregulin), pre-existing at the plasma membrane, as pro-ligand, by NTS has been proposed. These ligands are released by proteolytic cleavage involving enzymes of the metalloproteinase family, including ADAMS (disintegrin and metalloprotease) and MMP (matrix metalloproteinase; Sanderson et al., 2006; Zhao et al., 2007; Kataoka, 2009). Once released, these ligands bind to EGFR and activate the downstream signaling cascades of EGFR activation (Hassan et al., 2004). These results suggest a cooperative relationship between the neurotensinergic system and EGFR pathway.

Activation of MAPK pathway by NTS results in gene transcription stimulation, due to transcription factor activation, such as the induction of the early growth response gene-1 (Egr-1), the Ets family factors ELK1, and the AP-1 transcription factor family (Poinot-Chazel et al., 1996; Portier et al., 1998; Ehlers et al., 2000; Zhao et al., 2007). In colonic HCT166 cells, inhibition of PKC was shown to block NTS-induced DNA synthesis (Muller et al., 2011). In a tumor-initiating cell line derived from hepatocellular carcinoma (HCC) which is characterized by membrane expression of CD133, addition of exogenous NTS resulted in concomitant up-regulation of IL-8 and CXCL1 with simultaneous activation of MAPK and Raf-1, and promotion of angiogenesis, tumorigenesis, and self-renewal (Tang et al., 2012). The activation of MAPK via NTSR1 is mainly associated with uncontrolled cell growth, which can aggravate the growth of tumors (Harikumar et al., 2010; Kisfalvi et al., 2010).

Neurotensin also induces RhoGTPases and the non-receptor kinases focal adhesion kinase (FAK) and Src. Neurotensinergic system stimulation can modulate the activity of small RhoGTPases Rac1, Cdc42, and RhoA, which are partly responsible for the dynamics of the cytoskeleton. This modulation has an effect on cell migration. In the cell line U373 glioblastoma, the NTS has been associated with the stimulation of the activity of protein Rac1, RhoA, and Cdc42 (Zhao et al., 2003; Servotte et al., 2006). In addition, in small cell lung and prostate cancer cell lines, it has been shown that NTS can enhance the activity of focal adhesion kinase (FAK) (Tallett et al., 1996; Lee et al., 2001).

#### Neurotensin receptor 2, NTSR2

The low affinity receptor of NTS, NTSR2, is a protein of 410 amino acids, with a high homology to NTSR1 (64%) (Chalon et al., 1996). NTSR2 exhibits a low binding property for NTS and this binding can be inhibited by levocabastine, a non-peptide histamine H1 receptor antagonist (Schotte et al., 1986). SR48692, which has a lower affinity for NTSR2 than for NTSR1, can stimulate the activity of this receptor (Yamada et al., 1998). When the cloned mouse NTSR2 coding sequence is expressed in *Xenopus laevis* ovocytes, NTS, neuromedin N, levocabastine, and SR48692, are capable of triggering an inward current which is calcium-dependent (Mazella et al., 1996). Using CHO cells transfected with the cloned rat or human NTSR2 cDNA, levocabastine and SR 48692 can mobilize intracellular Ca^2+^ more intensively than NTS agonists and phosphorylate Erk1/2, suggesting that NTSR1 and NTSR2 receptors present distinct functional characteristics (Botto et al., 1997; Yamada et al., 1998; Gendron et al., 2004). In CHO cells transfected with human NTSR2 cDNA, both NTSR1 antagonists, SR48692 and SR142948A, enhance inositol phosphate (IP) formation with subsequent [Ca^2+^] immobilization, induce arachidonic acid release, and stimulate MAPK activity. Interestingly, these activities were inhibited by NTS and levocabastine in a dose-dependent manner. In summary, the signaling pathway triggered by NTSR2 is cell-dependent, and mainly based on its overexpression. This response is far different from that of the physiological endogenous expression.

#### Neurotensin receptor 3, gp85/sortilin, NTSR3

NTSR3 functions as a modulator of neurotensinergic signaling when it is co-expressed with another receptor of NTS, and as a functional receptor involved in the migration when expressed alone. This receptor is not NTS-specific. It can bind other ligands such as lipoprotein lipase, proneurotrophins, protein RAP (receptor-associated protein), or protein SAP (sphingolipid activator protein) (Nielsen et al., 1999; Lefrancois et al., 2003). NTSR3 may act as a co-receptor to participate in true NTS/NTSR1 signaling. The study by immunoprecipitation using the adenocarcinoma cell line HT29, demonstrated that the NTSR3 forms heterodimers with the NTSR1. Additionally, upon NTS stimulation, the NTSR1/NTSR3 complex is internalized and the interaction between the two receptors modulates both the NTS-induced phosphorylation of MAPK and the phosphoinositide (PI) turnover mediated by NTSR1 (Martin et al., 2002).

In the human microglial cell line C13NJ, NTSR3 is the only known endogenous NTS receptor. In these cells, NTS elicited cell migration by a mechanism dependent on both PI3K and MAPK pathways (Martin et al., 2003). The NTS/NTSR3 complex has been shown to phosphorylate both Erk1/2 and Akt kinases in a murine microglial cell line (Dicou, 2008).

## NEUROTENSIN/NEUROTENSIN RECEPTOR COMPLEX AND CANCER BIOLOGY

Few years after its discovery, high-level expression of NTS was found in the plasma of pancreatic tumor patients (Gutniak et al., 1980). This discovery inspired investigations on the relationship between NTS and cancer. Many studies have since been performed to clarify the role of NTS in carcinogenesis in diverse cancer cells.

### PANCREATIC CANCER

Pancreatic cancer is the eighth leading cause of cancer death in the world (Yabushita et al., 2012). It has the poorest prognosis amongst all human malignant solid tumors, mainly due to its high rate of metastasis (Cheng et al., 2012). The growth promoting action of NTS has been observed in pancreatic cancer cell lines both *in vitro* and *in vivo*. In both cases, the NTSR1 antagonist, SR48692, inhibited the NTS-induced effects (Sumi et al., 1993; Iwase et al., 1997). Recently, NTS was shown to protect insulin producing cells (b-TC3, INS-1E) against apoptosis induced by IL-1b and staurosporine (STS) (Coppola et al., 2008). NTSR2 and NTSR3 have been shown to be essential for the NTS mediated survival of these cells (Beraud-Dufour et al., 2009). NTS also influenced the migratory ability of pancreatic cancer cells, while NTS significantly reduced the migration levels of collectively migrating cells on vitronectin, NTS significantly increased the levels of individually migrating cells (Mijatovic et al., 2007). Thus, NTS-induced migration is dependent on the extracellular matrix environment and their respective migratory mode.

### COLORECTAL CANCER

Colorectal cancer is the third most common cancer worldwide and the fourth most common cause of death (Jemal et al., 2011). NTS stimulates the growth of mouse and human colon cancer cell lines in tissue culture and after being xenografted into nude mice (Maoret et al., 1999). *In vivo*, systemic NTS administration stimulates tumor size and weight, DNA, RNA, and the protein contents of the murine colon cancer, MC26 (Yoshinaga et al., 1992). Recently, the expression of NTSR1 was significantly correlated to an increase in the number of tumors when sporadic cancer was generated in mouse models by inflammation. However, no effect of NTSR1 expression was noticed on the number of aberrant crypt foci or tumor size, suggesting that the NTS/NTSR1 signaling complex has a major role in tumor progression (Bugni et al., 2012). NTS is also known to enhance colon cancer cell migration by increasing IL-8 expression and secretion. These effects are blocked by NTSR1 antagonists and curcumin, a diet-derived chemopreventive and/or chemotherapeutic agent which blocks AP-1 and NF-κB induction (Wang et al., 2006a). The role of IL-8 identified as an integral part of the metastasis process, was shown due to its triggering of the release of enzymes [MMPs, and uro-plasminogen activator (uPA)], responsible for extracellular matrix degradation (Xie, 2001).

### PROSTATE CANCER

Prostate cancer is the most common male malignancy in Western countries and the second most common cause of male cancer-related death in the UK and USA (Jemal et al., 2011). The androgen-dependent human prostate cancer LNCaP cell line has been shown to exhibit an autocrine growth response to NTS in androgen-deprived only conditions (Sehgal et al., 1994). Vias et al. (2007) found that long-term anti-androgen treatment of LNCaP cells produced a sub-line exhibiting upregulated expression of NTS and NTS receptors, which increased the proliferation rate, accelerated cell cycle progression, and increased invasiveness through Matrigel. These effects are sensitive to NTS siRNA. NTSR1 expression was found at very high levels in the human androgen-independent PC3 cell line, derived from prostate cancer metastasized to bone. The growth responses of these cells to NTS were found at concentrations close to human postprandial blood levels (Seethalakshmi et al., 1997). These studies proposed that NTS is a potential autocrine, paracrine, and endocrine regulator of prostate cancer growth in humans, after androgen ablation therapy and during the devastating final stages of the disease.

### LUNG CANCER

Lung cancer is the most common cause of cancer-related deaths throughout the world (Jemal et al., 2011). High concentration of NTS is present in and secreted from half classic small cell lung cancer cells (SCLC; Moody et al., 1985). NTS is one of the 73 genes overexpressed in the highly metastatic human lung cell line, H460-M, as compared to control cells (de Lange et al., 2003). In lung cancer cell lines, NCI-H209 and H345, SR48692 inhibited NTS-mediated calcium mobilization cells and c-fos mRNA induction and proliferation in a dose-dependent manner (Moody et al., 1985). SR48692 also inhibited tumor growth of NCI-H209 xenografts.

### BREAST CANCER

Breast cancer is the most common cancer in women, and the second leading cause of cancer deaths in women worldwide (Jemal et al., 2011). NTS immunoreactivity has been observed in breast cancers *in vivo*. NTSR1 expression has also been demonstrated in several breast cancer cell lines (Elek et al., 2000). The NTS anti-apoptotic effect has been described in the cell line MCF-7 originating from breast adenocarcinoma. Prolonged exposure to JMV449, a NTS-specific agonist, protected MCF-7 cells from serum deprivation-induced death, and reduced the number of apoptotic cells by two to three times. These effects have been predicted to be due, in part, to NTS-mediated induction of Bcl-2 mRNA and protein levels which depends on stimulation of MAPK (Somaï et al., 2002). By using NTSR1 Sh-RNA and SR 48692, tumor growth was significantly decreased when NTSR1 expression was abolished or blocked in experimental tumors of the breast (Souaze et al., 2006a).

## REGULATION OF THE NEUROTENSINERGIC SYSTEM

### REGULATION OF NTS

Neurotensin deregulation has been observed in many cancers such as in colonic adenocarcinomas, small cell lung carcinomas, non-small cell lung adenocarcinomas, medullary thyroid carcinomas, and in fibrolamellar HCCs (Baca and Schmidt-Gayk, 1981; Ulich et al., 1983; Moody et al., 1985; Dammrich et al., 1988; Ehrenfried et al., 1994). DNA methylation has been shown to play a crucial role in the expression of the gut endocrine gene neurotensin/neuromedin N (NT/N). In the human hepatoma cell line Hep G2 cells, methylation of a NT/N promoter construct resulted in a severe reduction of the promoter activity, whereas treatment with the demethylating agent 5-azacytidine-induced NT/N expression (Dong et al., 1998). These observations have been confirmed in different human colon cancer cell lines, either expressing or not the gene coding for NTS (Dong et al., 2000). Interestingly, NTS gene can be regulated by Ras while expressed. Both wild-type and activated Ras enhances expression of NTS in the gut-derived CaCo-2 cell line, by activating the proximal AP-1/CRE motif (Evers et al., 1995). More recently, the PI3K catalytic subunit, p110alfa, was demonstrated to negatively regulate NTS secretion *in vitro* and *in vivo*. This process involves several regulatory proteins such as α-tubulin deacetylase, small GTPase, and kinase D-interacting substrate (Li et al., 2012).

### REGULATION OF NTSR1

The mechanisms involved in NTSR1 deregulation in cancer cells have been studied in the context of colorectal carcinogenesis. These studies implicate an important role of the Wnt/β-catenin pathway deregulation. The mutation or loss of the protein APC (adenomatous polyposis coli) causes a dysfunction in the degradation of β-catenin. The accumulation of the latter in the cytoplasm, and its subsequent translocation to the nucleus induces NTSR1 gene expression via its association with transcription factors Tcf/Lef (T cell factor/lymphoid enhancing factor) (Souaze et al., 2006b). The NTSR1 promoter can be activated by the complex β-catenin/Tcf because it contains a consensus site for the transcription factors Tcf. In agreement with this result, it has been demonstrated that inhibitors of GSK-3β (protein kinase involved in the phosphorylation of β-catenin and its degradation) which cause the significant accumulation of β-catenin, upregulates the level of NTSR1 transcription (Wang et al., 2010). Similar results have been obtained in other cancers such as lung, prostate, and breast cancers (Chesire et al., 2002; Turashvili et al., 2006).

### REGULATION OF NTSR1 BY ITS OWN LIGAND

Upon acute agonist exposure, and under physiological conditions, initiated by β-arrestin-1 (βARR1), and β-arrestin-2 (βARR2), the NTS/NTSR1 complex is internalized and degraded in lysosomes through clathrin-coated vesicles. Cell resensitization occurs from *de novo* receptor synthesis a few hours after agonist removal (Souaze and Forgez, 2006; Law et al., 2012). However, some studies on cellular models such as the murine neuroblastoma cell line N1E-115 and human colon cancer cell line HT-29, showed a change in the traffic situation when the cell had a prolonged exposure to saturating doses of agonist (Souaze et al., 1997; Najimi et al., 1998). Instead of being degraded in the lysosome, NTSR1 accumulated transiently with NTS in the perinuclear recycling compartment (PNRC) where it was latter recycled to the plasma membrane (Toy-Miou-Leong et al., 2004). More recent research has shown the activity of endothelin-converting enzyme-1 (ECE-1) and βARRs being crucial for NTSR1 recycling and enhance NTS degradation (Law et al., 2012). Thus, NTS stimulation induces cellular adaptation by altering the degradation process of NTSR1. This phenomenon leads to permanently sensitizing cells to the neurotensinergic signal. The implementation of this mechanism could lead to deregulation of multiple signaling pathways involved in the cancer progression such as MAPK and its target genes.

## NEUROTENSIN/NEUROTENSIN RECEPTORS AND THERAPY

The implications of the previous sections suggest a more direct role for NTS/NTSR1 in cancer growth and progression, than has been previously attributed. Nevertheless, the ability to develop therapeutic strategies, around this complex, remain a challenge. Yet, despite them, the characteristics and qualities associated with this system should provide new pharmaceutical approaches as the system becomes further studied.

In the periphery and in the central nervous system, NTS mainly modulates the action of other molecules which are the principal effectors. Support for this view was confirmed by experiments with NTS- or NTSRs-deficient mice. These mice do not present any physiological disorder, are viable, and show normal growth and overt behavior. The deficient mice were also useful to implicate the NTS system in body temperature control (Pettibone et al., 2002), feeding regulation, weight control, and locomotion (Remaury et al., 2002). Mice were also less responsive or sensitive to exogenous agents or conditions. NTSR1 ko mice were less sensitive to the anorectic effects caused by leptin (Kim et al., 2008), and less responsive to morphine-induced analgesia (Roussy et al., 2010). Experiments with NTSR1- and NTSR2-deficient mice indicate a role in the regulation of ethanol consumption. However, NTSR1 regulates ethanol intoxication while NTSR2 is involved in ethanol-induced hypnosis (Lee et al., 2001, 2010). This suggests that the disruption of NTS system in the periphery is unlikely to generate side effects superior to the benefits expected in cancer treatment. On the other hand, the quasi exclusive alteration of the NTS system in tumors would confer an advantage in its use as a therapeutic target.

### BIOLOGICAL TOOLS USED TO INTERACT WITH THE NTS/NSTR SYSTEMS

Few biological tools have been developed to specifically target NTS system. The two most studied ones are the NTSR1 antagonists, SR 48692, and SR142948A (Gully et al., 1997, 1993). These molecules were developed to counteract NTS effects in central and peripheral nervous system. For this purpose, both molecules are specific and efficient. These compounds have been extensively used to successfully counteract exogenous NTS oncogenic actions. Nevertheless, these molecules remain weak and problematic to use to counteract intense autocrine regulation. For example, at high concentrations, SR48692 is toxic for the cells, and as no significant information exists on the biodegradability of these molecules, the dosage levels are unknown. In addition, SR 48692 acts as a low affinity agonist with NTSR2 (Yamada et al., 1998). NTRS2 expression also occurs in the gastric mucosa, in neuroendocrine cells of the stomach, and the small and large intestine, and in cells of the exocrine pancreas. NTSR2 is rarely detected in human-derived tumors (Schulz et al., 2006). Recently, NTSR2 was found overexpressed in B cell leukemia patient’s cells (Saada et al., 2012), and upregulated in prostate cancer cells luminal phenotype (Swift et al., 2010). It will be difficult to estimate NTSR2 impact on tumorigenesis until more information on its expression in human tumors becomes available.

In contrast, NTSR3 exhibits an ubiquitous expression in normal and tumoral cells. Its contribution on neoplastic progression is not known. The exact impact of SR48692 on NTSR3 remains complex. The most advanced hypothesis is that SR 48692 inhibits the oncogenic effects through NTSR1, when NTSR1 is expressed and in conjunction or not with NTSR3. In this context it was shown that SR 48692 inhibited the tumor growth of colon, breast, pancreas, and small lung cancer cells xenografted in mice (Iwase et al., 1997; Maoret et al., 1999; Moody et al., 2001; Souaze et al., 2006a). SR 48692 counteracts exogenously treated NTS in pancreas cells MIA PaCa-2 cells, bearing high and low affinity sites (Iwase et al., 1997), or circulating NTS as in the case of SW480, MDA-MB 231, and NCI-H209 cells (Maoret et al., 1999; Moody et al., 2001; Souaze et al., 2006a). It has to be noted that the use of SR 48692 does not stop tumor growth, but delays the growth rate by two to three times. The creation of the experimental tumor in mice results in tumors having an abnormal ratio (up to 10×) between the tumor size and body size, an observation uncommon and not seen in human tumors. Summarizing the effects induced by NTS, at the onset of cancer, NTS acts on the progression rather than on cellular transformation. Those actions, mediated by NTS include proliferation, survival, and metastatic effects. It is, therefore, not surprising to observe that the abolition of NTS/NTSR1 expression or signaling enhances the effect of several anti-tumoral treatments. For example, NTSR1 inhibition efficiently sensitized prostate regulation in orthotopic human tumor xenografts in mice to radiotherapy by significantly reducing tumor size, and in prostate cancer cells bearing NTS autocrine regulation (Valerie et al., 2011).

Another approach employed in the development of specific biological tools, uses Sh-RNA or Si RNA targeting NSTR1. The total or partial abolition of the target results in a decrease in cellular invasion, migration in HNSCC (Shimizu et al., 2008), or tumor growth of the human breast cancer cells (Souaze et al., 2006a). The two experimental designs demonstrate a contribution of NTS in these oncologic processes, through its interaction with NTRS1. At present, more knowledge is needed on the distribution and the coupling of NTSR2 in tumoral cells, as well as on the pharmaceutical tools specifically inhibiting NTSR2, to determine if NTSR2 is implicated in any oncogenic process.

### TARGETED APPROACHES FOR NTS/NTSR1

A recent approach under investigation is to use NTSR1-specific overexpression as a means to target the delivery of therapeutic or visualizing molecules in tumoral cells. This approach is made possible since NTSR1 endocytosis permits the introduction of molecules of interest inside the targeted cell and several strategies have been developed. In the first case, a non-viral gene transfer particle bearing six NTS molecules covalently bound to poly-lysine, enabled the transfer of a reporter and therapeutic gene to those tumor cells expressing NTSR1 when injected in the tumor or into the blood circulation of xenografted nude mice (Arango-Rodriguez et al., 2006). Interestingly, both injection sites showed a high proportion of transfected cells. In a second case, a therapeutic effect was also detected on experimental neuroblastoma tumors transfected with a thymidine kinase gene associated with ganciclovir. A severe reduction of tumor growth was also observed (Rubio-Zapata et al., 2009). In the same vein, a protein interfering with the cell synthesis machinery by inducing irreversible ADP-ribosylation of elongation factor 2, exotoxin A, was fused with NTS. This recombinant protein was able to specifically recognize NTSR1 positive cells and to exert a real *in vitro* cytotoxicity (Wick and Groner, 1997). In another strategy, oligo-branched peptides were used because of their proteolysis resistant nature. Tetra-branched NTS, armed with 5-FdU, increased *in vitro* and *in vivo* tumoral cell cytotoxicity as compared to the free drug (). The same idea was applied to carry the *cis*,*cis*,*trans*-diamminedichloridodisuccinatoplatinum(IV)–neurotensin bioconjugate specifically to tumor cells. The cytotoxicity of this molecule was tested on cell proliferation and found efficient when compared to the non-targeted platinum (Gaviglio et al., 2012).

Neurotensin-based radiopharmaceuticals have also been developed for tumor localization and therapy. Several strategies using different NTSR1 ligands were constructed whose main objectives were to limit the degradation of the molecule while maintaining the ligand specificity and a good affinity for NTSRs. Thus, the analog NT-XIX, with the three enzymatic cleavage sites stabilized showed a high specificity for NTSR1. A low accumulation of activity in the kidneys and a proper tumor-to-tissue ratio radioactivity clearance was observed *in vivo*, as well as a decrease in xenograft tumor growth (Garcia-Garayoa et al., 2009). More recently, new NTS analogs, DOTA-NT-20.3, and DOTA-NT-20.4 showed promising characteristics for imaging of NTS receptor-positive tumors and therapy (Alshoukr et al., 2009, 2011).

### NTSR1/NTS COMPLEX EXPRESSION IN HUMAN TUMORS

Several experiments have investigated the expression of NTSR1 in human tumors, but most of these studies remain too small or local. In normal colonic tissue, NTSR1 was not detected by immunohistochemistry, whereas in human colonic adenomas expression, NTSR1 was associated with cytoplasmic beta-catenin localization. This was one of the first suggestions that NTSR1 expression was an early event in colon carcinogenesis (Souaze et al., 2006b). NTSR1 mRNA expression studied by *in situ* hybridization showed a higher level of expression in adenocarcinoma as compared to adenomas. In addition, a higher intensity of NTSR1 expression was observed in filtrated adenocarcinomas into and beyond the muscularis propria as compared with tumors that were localized to the mucosa or submucosa, suggesting a contribution of NTS/NTSR1 complex in tumor expansion (Gui et al., 2008).

In pancreas, NTS binding sites were first studied by autoradiography, and found specifically in pancreatic cancer but not in normal pancreas and chronic pancreatitis. Nevertheless, northern blots showed NTSR1 mRNA in normal pancreatic cells, but an increase of transcript level in chronic pancreatitis and pancreatic cancer. Within the tumors, NTSR1 expression was higher in advanced stages as compared to early stages (Wang et al., 2000). NTSR1 and NTS protein expression was confirmed by immunohistochemistry in normal and tumoral tissue. In this latter case 80% expressed both NSTR1 and NTS (Wang et al., 2011).

More recently, a correlation between NTSR1 or NTS expression with outcome of the disease was evoked. In non-small lung cancer cells, NTS and NTSR1 expression were not detectable, whereas 60% of patients with stage 1 adenocarcinoma expressed either NTS or NTSR1 and 40% expressed both markers. Patients treated only by surgery did not receive adjuvant therapy and, in this cohort, NTRS1 expression was correlated with a worse prognosis of the disease (Alifano et al., 2010b). In contrast, NTS was found to be expressed in normal epithelial cells of breast tissue, whereas NTSR1 was absent. In breast invasive carcinomas high expression of NTSR1 was correlated with pejorative clinical parameters and disease outcome (Dupouy et al., 2009). In head and neck squamous cell carcinomas, NTS and NTSR1 mRNA high levels were significantly correlated with higher rates of distant metastasis as well as with the survival rate (Shimizu et al., 2008). In normal pleura, NTS and NTSR1 were found in 30 and 77%, respectively. In malignant pleural mesothelioma, expression increased to 71.1 and 90.4%, respectively. Interestingly in benign tissue, NTSR1 was located at the cell membrane whereas in tumoral cells, NTSR1 expression was granular and mainly restricted to the cytoplasm. The lack of presence at the cell surface suggested a state of permanent activation. In these cases, NTS high expression was associated with poor disease outcome (Alifano et al., 2010a).

### CONCLUSION

Cancer is more often regarded as a generalized disease and its treatment is mainly based on chemotherapy. In openly metastatic cancers, healing the sick is rarely achieved. In these cases only a prolongation of survival associated with a better quality of life can be expected. Therefore, it is necessary to improve our strategies. Recent treatment developments include the customization and selection of drugs specifically directed against biological and genetic abnormalities expressed by the tumor. However, as with conventional chemotherapy, these treatments are still insufficiently targeted and are often accompanied by serious side effects.

Two potential approaches (amongst others) to improve this situation include the highlighting of abnormalities specific to cancer cells, or the delivery of specific drugs against these pathological processes. This latter avenue would allow for effective treatment without side effects or benefit from a metabolic pathway in which it would be possible to connect conventional medicines and transfer them inside the cancer cells.

**FIGURE 1:**
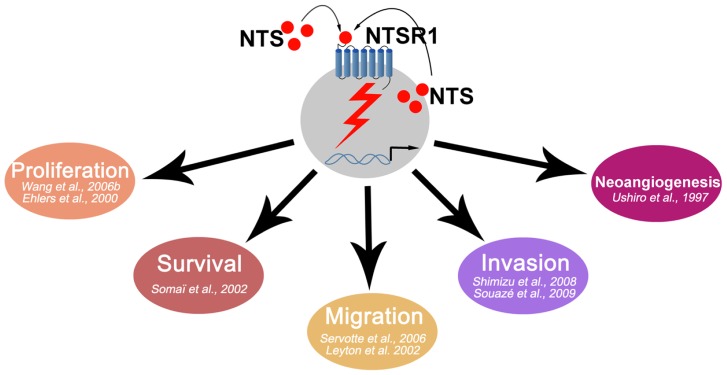
**Schematic representation of NTS oncogenic effects**.

The neurotensin and neurotensin 1 receptor system have provided insights for certain oncological situations. As seen above, NTS/NTSR1 is overactive in many tumors (**Figure 1**). The development of active antagonists, and those well tolerated by the patient, in human clinical trials will be a necessary step. NTS/NTSR1 may also be considered to become a carrier of chemotherapy drugs for tumor cells. In this regard, platinum salts, such as cisplatin, whose digestive and renal toxicity is well documented, are a prime target for the development of such tests. In all cases, the effectiveness of treatment can only be improved if the selection of patients is likely to benefit from such treatment. The last 45 years of work on the NTS/NTSR1 system have allowed for a better understanding of normal and cancer cells. This system can be further exploited for therapeutic purposes and for improving the management of cancer.

## Conflict of Interest Statement

The authors declare that the research was conducted in the absence of any commercial or financial relationships that could be construed as a potential conflict of interest.
